# Rodents of Bahir Dar Blue Nile River Millennium Park, Ethiopia

**DOI:** 10.1186/s40850-024-00216-w

**Published:** 2024-09-30

**Authors:** Dessalegn Ejigu, Marye Gelaw

**Affiliations:** Department of Biology, College of Science, Bahir Dar University, Bahir Dar, Ethiopia

**Keywords:** Diversity, Ethiopia, Relative abundance, Rodents, Trap success

## Abstract

**Background:**

Rodents are mammals of the order Rodentia, which are found in all continents except Antarctica. They are the most diverse groups of mammals representing 41% of all mammals and they are known with 33 families, 481 genera, and about 2277 species. The present study was conducted from December 2018 to August 2019 both during the dry and wet seasons using Sherman traps and live traps. Four trap sites namely forest, bushland, grassland, and sugarcane plantation were selected for data collection. Shannon-Wiener diversity index (H’) was used to compute rodent species diversity, Simpson’s index (D) to assess the number and abundance of rodents in the different habitats, and one-way ANOVA and independent samples t-test were used for data analysis.

**Results:**

A total of 163 individual rodents were captured in 1776 trap nights of which 125 individuals were captured using live traps in 1176 trap nights and 38 individuals using snap traps in 600 trap nights. Among the total individual rodents captured in the Bahir Dar Blue Nile River Millennium Park, 63.8% (*n* = 104) were males and 36.2% (*n* = 59) were females. Males outnumbered females in all the grids and the difference was significant (t = 31.5, df = 10, *p* < 0.05). The relative abundance of rodent species indicated that 42.4% (*n* = 53) were *Arvicanthis niloticus*, 26.4% (*n* = 33) *Rattus rattus*,* 17.6% (n =* 22) *Mus musculus*, 8% (*n* = 10) *Mastomys natalensis*, and 5.6% (*n* = 7) *Arvicanthis abyssinicus*. There are more individual specimens of *A. niloticus* than other species identified in the area (F = 698.22, df = 4, *p* < 0.05). Distribution of rodents varied among the different habitats. As a result, the bushland habitat showed more diversity of rodents (H = 0.98) and comprised relatively the highest (51.2%, *n* = 64) number of individuals captured, while the forest habitat supported the lowest number of rodents (4%, *n* = 5), and the difference showed a significant difference (F = 873.37, df = 3, *p* < 0.05). Population density with 95% confident interval estimated as 166 individuals/ha (95% CI:164.43–167.57).

**Conclusion:**

The present study provides basic information about population dynamics of rodents and contributes to design conservation strategies for rodents in particular and other biota of the area in general.

## Background

Rodents are the most diverse groups of mammals [1] with a nearly worldwide distribution [2, 3]. Habitats of rodents are highly diverse and they occur in every habitat from the Arctic Tundra to the Desserts [4]. They are among the most ubiquitous and numerous mammalian species [5]. Rodents are highly variable in morphology and behaviour [6]. They range from the tiny pigmy mice that weigh 5 g to the large capybaras that weigh up to 70 kg, from the arboreal flying squirrels to the subterranean mole rats, and from opportunistic omnivores to specialist feeders. Order Rodentia consists of 2277 species, 481 genera, and 33 families [7] and comprises 41% of the entire mammalian species [3]. However, they show less overall variation in their body plan than members of many other mammalian orders [2]. In Ethiopia, rodent fauna comprises more than 30% of the total mammalian species of the country [8]. Out of the 104 species of rodents in Ethiopia [9], 15 are endemic contributing to 50% of the total endemic mammalian fauna of the country. Family *Muridae* comprises 81% of the species and 93% of the endemic rodents [10]. Most rodent species play significant roles in maintaining the ecosystem including in seed dispersal, pollination, predator–prey relationship, and in maintaining ecological balance and habitat modification [11]. Population outbreaks of rodents occur when prolonged rainfall allows better survival of the young [12]. Climatic fluctuations and environmental variations are the driving factors for rodent outbreaks [13]. Thus, seasonal distribution and quantity of rainfall are considered major climatic variables in determining the population dynamics of rodents [14]. On the other hand, the absence of insufficient food and low ground cover largely determine the number of individual rodents leading to decreasing their diversity [15].

In Ethiopia, 11 species of rodents are major agricultural pests [13, 16]. Generally, rodents are opportunistic feeders, capable of changing their feeding habits depending on food availability. This generalist feeding behaviour of rodents makes them the most destructive agricultural pests [17]. Globally, rodents damage 30% of the crops during pre and post-harvest conditions [18]. Hence, monitoring the change in relative abundance of rodents is essential to conserve rodent diversity. Several new species of rodents have been discovered from different parts of the world and this revealed the need for further exploration of various habitats. In Ethiopia, studies on rodent diversity and abundance have been conducted in different part of the country. However, no previous studies were conducted in Bahir Dar Blue Nile River Millennium Park. Therefore, the main objective of this study is to determine diversity, relative abundance, and habitat association of rodents and it is useful to conserve rodent fauna in the area and to control rodent pests using appropriate rodent population management strategies.

## Results

### Species composition

A total of 163 individual rodents which belong to five different species were caught from a total of 1776 trap nights using live traps and snap traps. All the trapped individuals belonged to the family *Muridae*. Out of the total 163 captured individual rodents, 125 were trapped by live traps in 1176 trap nights and 38 individuals by snap traps in 600 trap nights. *Arvicanthis niloticus* was relatively the most abundant species followed by *Rattus rattus*, *Mus musculus*, *Mastomys natalensis* and relatively the lowest was *Arvicanthis abyssinicus.* There were more individuals of *A. niloticus* compared to other rodent species identified in the area and the difference was significant (F = 698.22, df = 4, *p* < 0.05) (Table 1).

**Table 1:** Species composition and relative abundance of live trapped rodent species (numbers in brackets indicate recaptures)

Common name	Scientific name	Total captured	Relative abundance (%)
African grass rat	*Arvicanthis niloticus*	53 (4)	42.4%
Black rat	*Rattus rattus*	33 (3)	26.4%
House mouse	*Mus musculus*	22 (2)	17.6%
Multi mammate rat	*Mastomys natalensis*	10 (2)	8%
Abyssinian grass rat	*Arvicanthis abyssinicus*	7 (2)	5.6%
	**Total**	**125 (13)**	**100%**

Rodent species diversity among the different habitats showed variations. Among the four habitat types, the bushland habitat comprised three species but the remaining three habitats harboured the same species richness each with two species. The bushland habitat was with high species diversity (H’=0.98), and Simpson’s index (D = 0.58) and the forest habitat was with the highest richness index (*R* = 0.62) (Table 2).

**Table 2:** Species richness and diversity of rodents in different habitats

Habitat type	N*o* of species	N*o* of captured	H’	D	*R*	
Forest	2	5	0.67	0.48	0.62	
Bushland	3	64	0.98	0.58	0.48	
Grassland	2	38	0.69	0.49	0.27	
Plantation	2	18	0.59	0.4	0.34	

H’=Shannon–Wiener diversity index, D = Simpson’s index, R = Richness index

### Distribution of rodents

The distribution of rodents varied among the different habitat types. The highest number of rodents were live-trapped from bushland habitat (51.2%, *n* = 64) which was followed by grassland habitat (30.4%, *n* = 38), and the lowest was obtained from forest habitat (4%, *n* = 5). There was a significant difference in rodent distribution among the different habitats (F = 873.37, df = 3, *p* < 0.05). *Arvicanthis niloticus* (42.4%) was the most widely distributed rodent species in the grassland and bushland habitats (Table 3).

**Table 3:** The distribution of rodents in different habitat types

Species	habitat types				Total catch (%)
	Forest	Bushland	Grassland	Plantation	
*A. niloticus*	-	35	18	-	42.4% (*n* = 53)
*R. rattus*	-		-20	13	26.4% (*n* = 33)
*M. musculus*	3	19	-	-	17.6% (*n* = 22)
*M. natalensis*	-	10	-	-	8% (*n* = 10)
*A. abyssinicus*	2	-	-	5	5.6% (*n* = 7)
**Total (%)**	**4% **(***n*** = **5**)	**51.2% **(***n*** = **64**)	**30.4% **(***n*** = **38**)	**14.4%** (***n*** = **18**)	**100%** (***n*** = **125**)

### Trap success

Trap success of rodents was varied among the different habitats or between seasons. Bushland habitat had the maximum (21.7%) trap success and the minimum (1.7%) was recorded in the forest habitat. Trap success was relatively higher among the four habitat types during the dry season than the wet season. However, the difference did not show a significant difference (t = 1.40, df = 6, *p* > 0.05). Capture rates varied from 3.4 to 29.2% (Table 4).

**Table 4:** Trap success during the dry and wet seasons among the four habitat types

Grid	Habitat type	Season	Total caught	Trap night	Trap success (%)
G1	Forest	dry	5	147	3.4%
		wet	-	147	-
G2	Bushland	dry	43	147	29.2%
		wet	21	147	14.2%
G3	Grassland	dry	27	147	18.3%
		wet	11	147	7.4%
G4	Plantation	dry	14	147	9.5%
		wet	4	147	2.7%
**Average trap success**					**12.1%**

### Age distribution of rodents

Age distribution of rodents between seasons showed that 44.8% (*n* = 56) and 16.8% (*n* = 21) of adults were caught during the dry and wet seasons, respectively, and the difference was significant (t = 38.67, df = 4, *p* < 0.05). There were more sub-adults 19.2% (*n* = 24) caught during the dry season that the wet season 8% (*n* = 10) with a significant difference (t = 16.92, df = 4, *p* < 0.05). Juveniles had the lowest number of individuals during the dry (7.2%, *n* = 9) and wet (%, (*n* = 5) seasons, respectively, and the difference showed significant differences (t = 7.33, df = 4, *p* < 0.05) (Table 5).

**Table 5:** Age distribution of live trapped rodents

Species	Number of individual age groups of rodents						Total
	Adult		Sub adult		Juvenile		
	dry	wet	dry	wet	dry	wet	
*A. niloticus*	23	11	10	4	3	2	53
*R. rattus*	16	5	6	3	2	1	33
*M. musculus*	9	3	4	2	3	1	22
*M. natalensis*	4	2	2	1	1	-	10
*A. abyssinicus*	4	-	2	-	-	1	7
**Total**	**56**	**21**	**24**	**10**	**9**	**5**	**125**

### Population density

The overall population density of rodents in Bahir Dar Blue Nile River Millennium Park was estimated based on the population numbers of rodent species in the live trapping grids using the Minimum Number Alive (MNA) method with 95% confident interval, and the result showed that there were 166 individuals/ha (95% CI:164.43–167.57). Concerning habitat, the highest estimated population density of rodents was recorded from the bushland (84/ha) which was followed by grassland habitat (50/ha), and the lowest was in the forest habitat (7/ha). Population density of rodents among the different habitats showed a significant difference (F = 1873.63, df = 3, *p* < 0.05). Among the species of rodents in the area, the overall population density of *A. niloticus* was the highest (70/ha) and the lowest was *A. abyssinicus* (11/ha) (Table 6).

**Table 6:** Population density estimate of rodents in different habitats

Species		Habitat types				Total
	Seasons	Forest	Bushland	Grassland	Plantation	
*A*. *niloticus*	dry	-	30	17	-	47
	wet	-	16	7	-	23
*R*. *rattus*	dry	-	-	18	13	31
	wet	-	-	8	4	12
*M*. *musculus*	dry	4	17	-	-	21
	wet	-	8	-	-	8
*M*. *natalensis*	dry	-	9	-	-	9
	wet	-	4	-	-	4
*A. abyssinicus*	dry	3	-	-	6	9
	wet	-	-	-	2	2
	**Total**	**7**	**84**	**50**	**25**	**166**

### Snap trapping

A total of 38 individual rodents were captured with a trap success of 6.3% using snap traps during the dry and wet seasons in 600 trap nights. Hence, five species of rodents were trapped and the highest number of rodents were snap trapped in bushland habitats (50%, *n* = 19) and the lowest was in forests (5.2%, *n* = 2), and there was a significant difference among snap trapped rodents among the different habitats (F = 172.43, df = 3, *p* < 0.05). Regarding species relative abundance, *A. niloticus* was the highest (34.2%, *n* = 13) and *A. abyssinicus* (7.9%, *n* = 3) was the lowest (Table 7).

**Table 7:** Species composition and relative abundance of snap trapped rodents

Habitat	Trapped species					
	A. niloticus	*R*. rattus	M. musculus	M. natalensis	A. abyssinicus	Total (%)
Forest	-	-	1	-	1	5.2%, *n* = 2
Bushland	7	-	7	5	-	50%, *n* = 19
Grassland	6	5	-	-	-	29%, *n* = 11
Plantation	-	4	-	-	2	15.8%, *n* = 6
**Total**	**34.2%**, ***n*** = **13**	**23.7% ***n* = **9**	**21%**, ***n*** = **8**	**13.2%**, ***n*** = **5**	**7.9%**, *n* = **3**	**100%**, *n* = **38**

Body weight and body measurement of snap-trapped rodents showed that *A. niloticus* has relatively the heaviest mean body weight of 87.9 ± 5.2 g during the wet season, while the lightest body weight (53.6 ± 7.7 g) was obtained from *A. abyssinicus* during the dry season. However, there was no significant difference in mean body weight among species (F = 2.36, df = 3, *p* > 0.05) (Table 8).

**Table 8:** Mean body weight (g) and measurements (cm) of snap trapped rodents

Species	Season	No,	Body Measurement				
			BW [g]	HB [cm]	TL [cm]	HF [cm]	EL [cm]
A. *abyssinicus*	dry	5	53.6 ± 7.7	13.0 ± 0.7	14.5 ± 1.5	2.8 ± 0.3	2.3 ± 0.5
	wet	8	65.1 ± 10	12.3 ± 0.9	15.2 ± 1.4	2.6 ± 0.4	2.0 ± 0.4
A. *niloticus*	dry	10	68.8 ± 4.4	12.5 ± 0.3	8.9 ± 0.5	2.4 ± 0.3	1.2 ± 0.2
	wet	7	87.9 ± 5.2	14.2 ± 0.5	10.3 ± 0.4	3 ± 0.2	1.3 ± 0.2
*M. musculus*	dry	4	63.5 ± 4.2	12.7 ± 0.8	18.3 ± 0.9	3.7 ± 0.3	2.5 ± 0.3
	wet	3	73.3 ± 8.5	15.8 ± 2.3	17.5 ± 1.2	3.5 ± 0.2	2.3 ± 0.2
*M. natalensis*	dry	2	76.5 ± 2.5	13.0 ± 0.5	13.3 ± 0.3	2.5 ± 0.1	1.4 ± 0.1
	wet	2	63.5 ± 5.2	15.5 ± 0.8	15.9 ± 0.6	28.8 ± 0.3	1.5 ± 0.2

BW = Body Weight, HB = head-body length, TL = tail length, HF = Hind foot length and EL = ear length

### Reproductive condition

The number of embryos of pregnant female rodents varied among the different species and between seasons in the same species. More pregnant females were caught during the wet than the dry seasons. Sixteen pregnant female rodents grouped into four species were captured using snap traps. During the wet season, a greater number of embryos were recorded in *M. natalensis*. The number of embryos observed in the snap trapped females ranged from 3 to 5 during the dry season and 6–9 during the wet season, and more embryos were counted during the wet season than the dry season. However, the difference was not significant (t = 1.40, df = 6, *p* > 0.05) (Table 9).

**Table 9:** Number of embryos recorded from snap trapped pregnant females

Species	Season	Number of dissected females	Number of embryos
*M. natalensis*	dry	1	5
	wet	3	9
*A. abyssinicus*	dry	2	5
	wet	2	6
*A. niloticus*	dry	2	6
	wet	4	7
*R. rattus*	dry	1	5
	wet	1	6

## Discussion

A total of five species of rodents were identified from 125 live-trapped rodents in Bahir Dar Blue Nile River Millennium Park. The highest numbers of rodents were trapped in bushland habitats. The findings of [16] showed that more diversity of rodents was caught in the natural ecosystem than in modified ones. Similarly [19, 20], reported six species in the Menagesha State Forest and the Bir farm, respectively, and [21], seven species from the Bale Mountains. Thus, rodent species composition in the present study area is comparable to other parts of the country, Ethiopia. However, it is the lowest compared to the findings of [22] in the Aquatimo forest patches and adjacent farmland. The similarity of rodent diversity in different parts of Ethiopia might indicate the occurrence of similar degraded habitats mainly caused by various anthropogenic activities including overgrazing by livestock, deforestation, and agricultural expansion into the natural habitats. *Arvicanthis niloticus* was the most abundant species in the present study area, which was similar to the findings of [23, 24]. In Ethiopia, the distribution of *A*. *niloticus ranges* from sea level to 2000 m a.s.l [25]. In the present study area, *A. niloticus* was trapped in the grassland and bushland habitats. Relatively, the lowest in abundance was *A. abyssinicus*. However, *A. abyssinicus* is a common species in the Ethiopian Plateau [25, 26] where more individuals of this species were recorded in the cultivated habitats. Habitat complexity in food availability and cover is a key factor in influencing the overall distribution of rodents in the present study area. According to [27] there is an association between small mammals’ composition and availability of resources in the area. As a result, the habitat association of rodents in the Bahir Dar Blue Nile River Millennium Park showed variations in diversity and abundance of species across the different habitat types. Thus, the relative abundance of rodent species showed differences among the different habitat types with the highest in the bushland habitat as it supports three species, while the sugarcane plantation, grassland, and forest habitats each support two species. The bushland habitat is characterized by diverse and densely grown medium sized vegetation and provides sufficient food, shelter, and cover to escape from predators. The findings of [28] confirmed that bushy habitat provides safe sites for germination and growth of herbaceous vegetation, thereby enhancing the diversity of food resources for small mammals. However, the lower abundance of rodents in the forest habitat might be a result of sparse ground vegetation cover that might expose them to predation.

The present research findings showed variation in the total catch and trap success of rodents between the dry and wet seasons. Seasonality might cause dynamic changes in the habitats including vegetation cover, rainfall, and food availability [12]. During the dry season, the grasses which cover the grassland habitat are overgrazed by livestock and the remaining grasses are cut down for forage by the local community. During the wet season, the grassland habitat becomes swampy and it might not be suitable for grassland inhabited rodents. As a result, the rodents are forced to migrate to the nearby bushland habitats where enough food and shelter are available. Consequently, the grassland habitat harbours a lower number of rodents compared with the bushland habitat both during the dry and wet seasons.

The number of adult rodents trapped by live trapping method was higher than sub-adults and juveniles. Out of the total rodents trapped using live and snap trapping techniques during the dry and wet seasons, males comprised 63.8% and 36.2% were females. This shows that the number of males was higher than females. Trap success varied from habitat to habitat and between seasons. Trap success during the dry and wet seasons was 15.1% and 6.07%, respectively. The average trap success for the two seasons was 10.6%, which was slightly lower than the trap success of 11.5% reported by [29] in the maize farms in Ziway area and 18.7% by [21] in the Bale Mountains. These differences in trap success among the different habitats might be due to differences in quality of habitats since the habitats in the Blue Nile River Millennium Park are highly degraded due to overgrazing by livestock and various anthropogenic disturbances compared with the maize farms and intact habitats in the Bale Mountains National Park.

Rodents’ population varies between trapping seasons and a higher rodent population was trapped during the dry season than the wet season which contradicts with the findings of [30] in the Wonji sugarcane area since in this area the highest rodent population was trapped during the wet season than the dry season. However, the findings of the present research are in line with the results of different researchers [20, 31, 32]. Different environmental factors including quality and abundance of food, weather conditions, and predation cause population fluctuations in rodents [33].

More pregnant females were caught during the wet season than the dry season regardless of the higher rodent population trapped during the dry season compared to the wet season. This result is also in line with the findings of [19, 31, 34, 35]. This is because, during the wet season, the rainfall contributes to the availability of other ecological requirements needed to support the breeding of females and the newborn infants. Pregnant *R. rattus* was trapped both during the dry and wet seasons indicating that this rodent species can breed throughout the year. In both seasons, pregnant females of *M. natalensis* were trapped both during the dry and wet seasons although they were more in number during the wet season. This might be evidence of the continuous breeding behaviour of rodents that occur throughout the year [32]. However, the findings of other researchers indicated that *M. natalensis* showed a strict breeding seasonality that is closely associated with the availability of rain [17]. Species of *Arvicanthis* and *Mastomys* are the most important agricultural pests in Africa [32], and they are widely distributed in the tropical parts of Africa. According to [13], *Arvicanthis* and *Mastomys* are major pests in the maize croplands of Ethiopia. Periodic population explosions of pest species are a particular hazard for rural farmers in many parts of Africa [2]. Thus, there is a need to monitor the population dynamics of rodents to ensure their conservation as well as to control rodent pests in the area.

## Conclusion

The present study provides valuable information on diversity, relative abundance, and habitat association of rodent species in Bahir Dar Blue Nile River Millennium Park. Variations in abundance among the rodent species were observed in all habitats and between seasons. Therefore, there is a need to protect the entire habitat of the park to ensure the conservation of rodents in particular and other small mammalian species in general. More pregnant females were caught during the wet season than the dry season indicating that breeding in rodents is associated with rainfall that can influence the availability of other ecological requirements of rodents.

The natural ecosystems of the Blue Nile River and the forest and bushland habitats coverage of the Bahir Dar Blue Nile River Millennium Park have changed from time to time because of various anthropogenic activities including deforestation, agricultural expansion, and cutting of trees for firewood. These human disturbances could impact the diversity and abundance of rodents in particular and other faunal diversities of the park at large. Therefore, the local community who settled around the park should take the responsibility to conserve the park’s biodiversity. Moreover, the government of the Amhara Region should implement appropriate conservation measures including strict law enforcement mechanisms to protect the entire ecosystems of the area.

## Methods

### Description of the study area

**Fig. 1:**
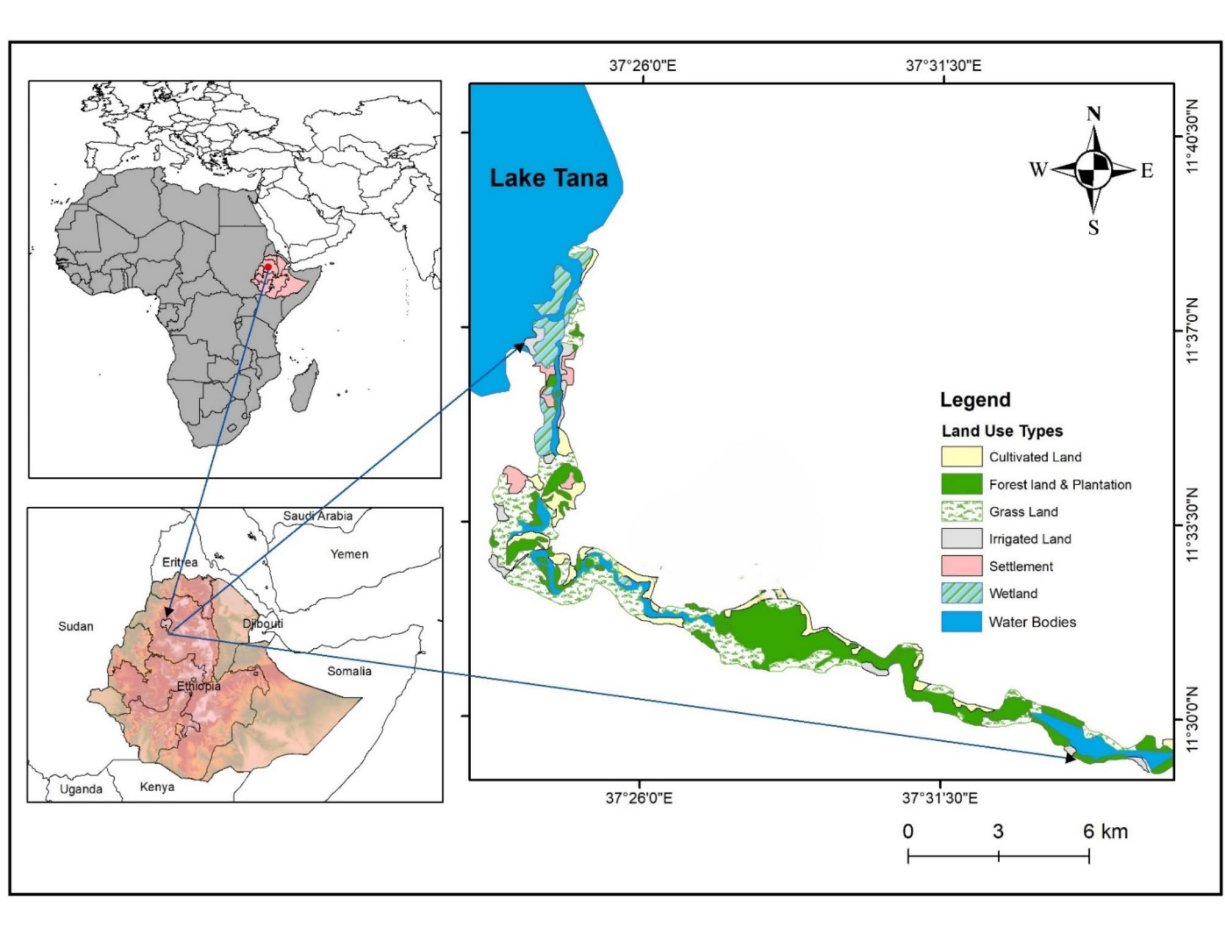
Location map of the study area

This study was conducted in Bahir Dar Blue Nile River Millennium Park, which is located between 11^o^29^’^40.2^’’^ – 11^o^37’29.9’’N latitudes and 37^o^24^’^37.2^’’^ – 37^o^36’34.0’’E longitudes (Fig. 1). The park extends from the head of the Blue Nile River at the southern tip of Lake Tana to Tis-Isat Falls on either side of the Blue Nile River, which is the largest river in terms of volume of discharge in Ethiopia [36]. Tis-Isat, which is located about 25 km from Lake Tana, is a significant waterfall where the river drops 50 m into the Blue Nile Gorge. The park covers about 4680 ha [37]. The ten year (2009 to 2019) mean monthly maximum and minimum temperatures recorded at Bahir Dar Meteorological Station are 30.2^o^C and 7.9^o^C, respectively. Rainfall data of the area shows uni-modal distribution and the ten years annual average rainfall of the area is 1461.2 mm with the highest rainfall from June to September. The study area supports a diverse group of plant and animal species. The forest habitat is dominated by different indigenous woody species including *Syzygium guineense*, *Mimusops kummel*, *Acacia spp*., *Albizia schmperiana*, *Apodytes dimidiata*, *Brucea antidysenterica*, *Combretum molle*, *Juniperus procera*, *Olea europaea*, *Osyris quadripartite*,* Croto macrostachyus*,* Ficus spp.*,* Millettia ferruginea* and *Podocarpus falcatus*. The grassland habitat is dominated by different types of grass species including *Cyperus papyrus*, *Trifolium pretense*, *Bidens pilosa*, *Pennisetum romosum* and *Cynodon dactylon*. The farmland habitat (plantation) is a modified habitat used to cultivate different annual and perennial species mainly sugarcane.

### Data collection methods

A preliminary survey was conducted at the beginning of December 2018 to gather all the relevant information about the area. The study area was classified into four habitats namely forest, bushland, grassland, and cultivated land (sugarcane farm). To collect data on rodents, the representative grids were established randomly among the different habitat types. Most studies of rodents rely on sampling methods involving trapping with space ranging from 5 to 20 m which is a typical range for small mammals [38]. Trapping grids are more structured in arrangements with traps set in a parallel line that ensures an even density of traps per unit area.

Sampling sites constituted an area of 3600 m^2^ and 49 Sherman live traps and 25 snap traps were used for data collection during the dry and wet seasons [39]. Data for diversity, relative abundance, and habitat association of rodents in different habitats within the Blue Nile River Millennium Park were collected using standard trapping techniques. To reduce the overlapping of captures, all live trapping grids were separated at about 500 m apart. Snap traps were used for stomach content analysis and embryo count. Data for the dry season were collected from January to March 2019 and the wet season data were obtained from June to August 2019.

### Data collection using live-traps

Live traps of 49 in number were set at intervals of 10 m into horizontal and vertical directions forming 60 × 60 m (3600m^2^) with seven rows and seven columns in 7 × 7 arrangements (Table 10). This trapping design is referred to as the standard grid [40]. The traps were used to capture live rodents from each of the four habitat types (forest, bushland, grassland, and cultivated land) to determine the species richness, abundance, and habitat association of rodents.

Each trap location was marked with coloured plastic tags on the visible part of the nearby tree and in the grassland grids; the tallest grasses were tied at each trapping station to locate the traps easily. The traps baited with peanuts were covered by grass and plant leaves during the dry seasons to protect against strong heat and extreme cold and checked twice a day in the morning (07:00–08:00 a.m.) and late in the afternoon (05:00–06:00 p.m.) for three consecutive nights and days each during the dry and wet seasons to clean and re-bait the traps.

**Table 10:** Diagrammatic representation of Sherman live trapping grids

A1	B1	C1	D1	E1	F1	G1
A2	B2	C2	D2	E2	F2	G2
A3	B3	C3	D3	E3	F3	G3
A4	B4	C4	D4	E4	F4	G4
A5	B5	C5	D5	E5	F5	G5
A6	B6	C6	D6	E6	F6	G6
A7	B7	C7	D7	E7	F7	G7

### Data collection using snap-traps

Snap traps were baited with peanut butter and set along with Sherman live traps to collect data on body weight, sex, appropriate age, reproductive status, and standard body measurements in each type of habitat both during the dry and wet seasons. Snap traps were arranged at 500 m away from the live traps but 20 m away from each other for three consecutive days and checked twice a day early in the morning and late in the afternoon hours after the live traps had been checked (Table 11).

**Table 11:** Diagrammatic representation of snap –trapping grids

a1	b1	c1	d1	e1
a2	b2	c2	d2	e2
a3	b3	c3	d3	e3
a4	b4	c4	d4	e4
a5	b5	c5	d5	e5

Data on the approximate age, sex and reproductive status of rodents were recorded using snap traps. According to [20], the approximate ages of rodents were determined based on weight and pelage colour. Sex was determined in males by the presence of testes, a penis, and a larger anogenital distance, but in females by the presence of nipples, a vagina, a clitoris, and a shorter anogenital distance. Reproductive status in females such as closed or perforated vagina were determined using their enlarged nipples, large swollen abdomen and body weight, and presence of a copulatory plug in reproductively active females. Reproductive status in males was detected by the colour and position of testicles and scrotal [15]. Trapped rodents were removed from the traps and placed in a polythene bag, weighed using spring balance, identified, marked by toe clipping, and released back to the site from where it was trapped [40]. Voucher specimens were taken to the Zoological Natural History Museum of Addis Ababa University for comparison with already available Museum specimens.

### Data analysis

Shannon-Weaver diversity index was used to evaluate the diversity of rodent species in different habitats within the study area and calculated as H’= -∑ [(pi) ln(pi)], where ‘pi’ refers to the proportion of species ‘i’ in the sample (the relative abundance of that species [Ni/N_tot_]). Simpson’s index (D) was used to assess the diversity and abundance of the different habitats concerning the presence of rodents. Species richness of rodents in different habitats was determined using the number of captured at each grid and the richness index (R). Capture Percentage (CP) refers to the abundance of each species relative to the habitat type concerning the total capture and it was calculated as (CP) = (Ni/N_tot_) ×100, where, the Ni = number of individuals of each species in each habitat, N_tot_= total number of individuals caught during the entire study period. Populations of rodents during the different trapping sessions were estimated by the Minimum Number Alive (MNA) estimation method with 95% confidence interval. The relative abundance of rodents was assessed as the percentage of trap success between the dry and wet seasons and habitat types. Trapping Success (TS) was calculated as (TS) = (Ni/Tn) ×100 where, Ni = the number of individuals of each species, Tn = the total number of trap nights. TS tells how many of the traps set at a site were able to capture the target species. One-way ANOVA and independent samples t-test were used for data analysis. Statistical tests were carried out using SPSS software version 20 with a significant level of *p* < 0.05.

## Data Availability

All data generated and analyzed during this manuscript preparation are available on the hands of the corresponding author.
